# Protein translation paradox: Implications in translational regulation of aging

**DOI:** 10.3389/fcell.2023.1129281

**Published:** 2023-01-13

**Authors:** Harper S. Kim, Andrew M. Pickering

**Affiliations:** ^1^ Center for Neurodegeneration and Experimental Therapeutics (CNET), Department of Neurology, Heersink School of Medicine, University of Alabama at Birmingham, Birmingham, AL, United States; ^2^ Medical Scientist Training Program, Heersink School of Medicine, University of Alabama at Birmingham, Birmingham, AL, United States

**Keywords:** protein translation, aging, ageing, lifespan, hallmarks of aging, sk6, eIF, theories of aging

## Abstract

Protein translation is an essential cellular process playing key roles in growth and development. Protein translation declines over the course of age in multiple animal species, including nematodes, fruit flies, mice, rats, and even humans. In all these species, protein translation transiently peaks in early adulthood with a subsequent drop over the course of age. Conversely, lifelong reductions in protein translation have been found to extend lifespan and healthspan in multiple animal models. These findings raise the protein synthesis paradox: age-related declines in protein synthesis should be detrimental, but life-long reductions in protein translation paradoxically slow down aging and prolong lifespan. This article discusses the nature of this paradox and complies an extensive body of work demonstrating protein translation as a modulator of lifespan and healthspan.

## 1 Introduction

**FIGURE 1:**
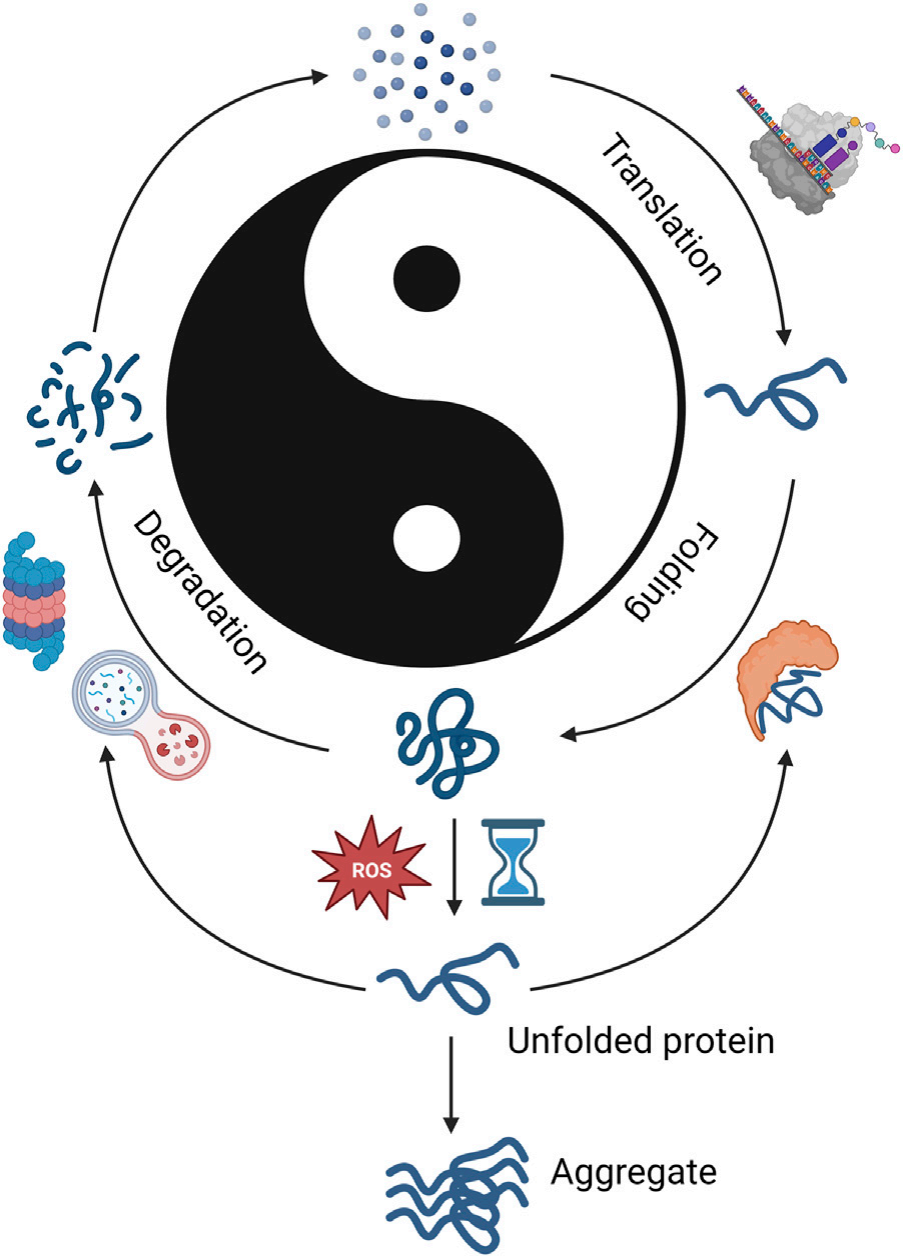
Protein lifecycle. Graphical representation of proteostatic cycle. Protein synthesis is shown on the right and protein degradation on the left. The Chinese philosophical concept Yin and Yang are denoted in the center with Yang denotating synthesis and Yin degradation. This is used to denote the importance of balance between synthesis and degradation. This figure shows that in balanced proteostasis, proteins are translated using the available amino acid pool (top right), folded by molecular chaperones (bottom right), degraded by proteasome or autophagial pathways (bottom left), and amino acids are recycled to form new proteins. Imbalance from insufficient molecular chaperone function, ROS, or insufficient degradation leads to unfolded proteins (bottom center), which may be refolded by molecular chaperones or removed by degradation pathways. If protein quality control does not occur, aggregates may form. Created with BioRender.com.

The life of a protein begins with translation of mRNA into a nascent polypeptide chain [reviewed in (Hebert and Molinari 2007; Taylor and Dillin 2011; Balchin et al., 2016)]. During or after synthesis, a protein then adopts a higher-level stable three-dimensional structure to become biologically functional. This process is called co- or post-translational folding and occurs spontaneously; only the information contained in the amino acid sequence is required for folding of proteins into their native state. Especially, chemical forces created by specific amino acid sequences within the particular position of the polypeptide chain (e.g. hydrophobic interactions and hydrogen bonds) guide the proper folding. Although protein folding can occur spontaneously, there are several molecular chaperones that facilitate the folding by accelerating rate-limiting steps. Chaperones also assist refolding of unfolded or denatured proteins and prevent them from being aggregated. Once a protein is properly folded, it is chemically modified and trafficked to the correct cellular compartment. Finally, when proteins reach the end of their life or become damaged, they get degraded by the autophagy-lysosomal pathway or ubiquitin-proteasome system (UPS) (Figure 1).

Disruption in any of the process mentioned above can disturb protein homeostasis (proteostasis), cause protein aggregations and cellular death, and ultimately contribute to the pathogenesis of several diseases, including neurodegenerative disorders (Hebert and Molinari 2007; Powers et al., 2009; Taylor and Dillin 2011).

To date, a large body of research has demonstrated dysfunction in many of aspects of the proteostatic network as a critical component of aging. The proteasome system was shown to deteriorate as a consequence of either age or disease; conversely, enhancing proteasome function can extend lifespan/healthspan and protect against age-related diseases (Munkacsy et al., 2019; Nguyen et al., 2019; Chocron et al., 2022). In addition, several studies demonstrated dysfunction of autophagial pathway with age and how augmentation of the autophagial system can extend lifespan (Hansen et al., 2018). Likewise, functions of molecular chaperones were shown to decline with age, whereas overexpression of such chaperones was able to extend lifespan (Soti and Csermely 2003). Despite extensive aging studies in degradation systems, there is a lack of studies in the other arm of the proteostatic control: protein synthesis. In this review, we extensively discuss how modulation of protein synthesis and lifetime protein translation dynamics regulate the onset of aging.

## 2 The process of eukaryotic protein translation

**FIGURE 2:**
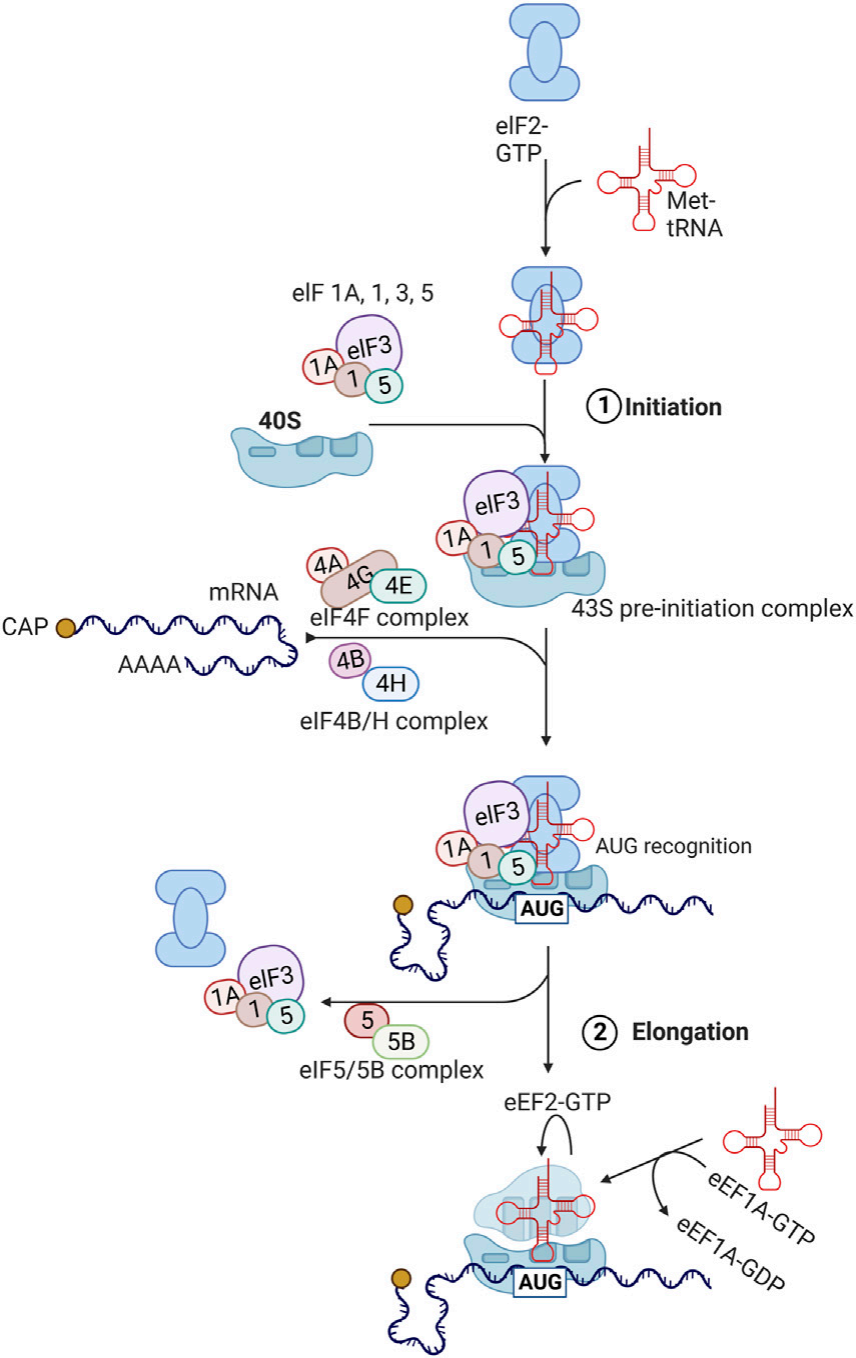
Protein translation pathways. The process of translation initiation and elongation in eukaryotic cells. Key eukaryotic initiation factors (eIFs) and eukaryotic elongation factors (eEFs) are highlighted. This process is described in detail in section 3 with involvement of highlighted factors in aging described in section 5. Created with BioRender.com.

Protein translation occurs in three phases: 1) initiation of mRNA translation, 2) elongation of the polypeptide chain, and 3) termination of mRNA translation (Sonenberg and Hinnebusch 2009; Jackson et al., 2010) (Figure 2).

During the initiation phase, the eukaryotic initiation factor 2 (eIF2) forms a ternary complex with GTP and methionine-charged methionyl-tRNA (Met-tRNA_i_^Met^) (Sonenberg and Hinnebusch 2009; Jackson et al., 2010). The ternary complex then binds to the small 40S ribosomal subunit along with several eukaryotic initiation factors (eIF1, eIF1A, eIF3, and eIF5) to form the 43S pre-initiation complex. Subsequently, eIF4F complex, consisting of eIF4E, eIF4G, and eIF4A binds to the 5′ cap region of mRNA and unwinds this region in an ATP-dependent manner with eIF4B. This allows for the 43S pre-initiation complex to attach to the mRNA being translated. The 43S complex then scans the 5′untranslated region (5′ UTR) from the 5′ to 3′ direction to the initiation codon (AUG). After the AUG codon is recognized, eIF2-bound GTP is hydrolyzed by eIF5 and eIF5B, leading to dissociation of eIFs and joining of 60S ribosomal subunit to form the elongation-competent 80S ribosome.

Elongation of peptide chains by the 80S ribosome is assisted by eukaryotic translation elongation factors (eEFs) (Sonenberg and Hinnebusch 2009; Jackson et al., 2010). Once aminoacyl tRNA synthetase loads the tRNA with the amino acid corresponding to the codon, eEF1A-GTP complex brings this incoming aminoacyl-tRNA to the A site of the ribosome. If the correct aminoacyl-tRNA fits to the A site, GTP is hydrolyzed and eEF1A-GDP complex becomes dissociated. eEF1B catalyzes the exchange of bound GDP for GTP, enabling the next cycle to occur again. Then, peptidyltransferase in the 60S ribosomal subunit (28S ribosomal rRNA) catalyzes the transfer of peptide attached to the aminoacyl-tRNA in the P site to the amino group at the aminoacyl-tRNA in the A site, forming a peptide bond. Subsequently, eEF2-GTP complex facilitates the transfer of peptidyl-tRNA from the A site to the P site, which is called translocation.

Finally, when the A site encounters a stop codon (UAG, UAA, or UGA), releasing factor (eRF) binds GTP and stimulates the transfer of peptidyl group from the P-site tRNA to H_2_O instead of the A-site tRNA (Sonenberg and Hinnebusch 2009; Jackson et al., 2010). This results in the release of the peptide along with uncharged tRNA and eRF-GDP complex, followed by dissociation of 40S and 60 S ribosomal subunits.

## 3 Regulation of protein translation rate

The first initiation phase of protein translation is a rate limiting step, where most regulations are exerted (Gebauer and Hentze 2004; Sonenberg and Hinnebusch 2009; Jackson et al., 2010). The global rate of protein translation is mostly regulated by altering the activity and availability of eIFs. Two most heavily regulated eIFs to adjust the rate of protein translation are eIF2 and eIF4E.

eIF2 consists of three subunits: α, β, and γ (Gebauer and Hentze 2004; Sonenberg and Hinnebusch 2009; Jackson et al., 2010). GTP hydrolysis by the γ subunit of eIF2 facilitates the formation of ternary complex, which is the key step in translation initiation. Exchange of GDP for GTP is catalyzed by eIF2B and is necessary for regenerating active eIF2. Phosphorylation of the α subunit of eIF2 impedes the dissociation of eIF2B, blocks the GDP-GTP exchange, and thereby pronouncedly lowers the rate of protein translation (Donnelly et al., 2013). As such, eIF2α phosphorylation by eIF2α kinases is frequently used by cells to attenuate protein translation in response to physiological or environmental conditions. For example, when unfolded or misfolded proteins accumulate in the lumen of endoplasmic reticulum (ER), one of the eIF2α kinases called PKR-like ER kinase (PERK) phosphorylates eIF2α, reduces the protein translation rate, and thereby decreases the ER protein folding load (Walter and Ron 2011). This translational control by PERK is one of the branches of the so-called unfolded protein response (UPR) (Walter and Ron 2011). Another kind of eIF2α kinase called GCN2 is primarily activated by amino acid and glucose deprivation and lowers the rate of protein translation to adjust for nutrient availability (Zhang et al., 2002a; Donnelly et al., 2013).

The cap binding protein eIF4E facilitates the recruitment of the 43S pre-initiation complex to the 5′cap, which is regarded as the rate-limiting step in protein translation initiation (Jackson et al., 2010). The availability of active eIF4E is regulated by the eIF4E-binding protein (4E-BP) (Marcotrigiano et al., 1999; Musa et al., 2016). 4E-BP prevents eIF4E from interacting with eIF4G, thereby blocking the formation of eIF4F complex and halting protein translation initiation. The ability of 4E-BP to bind eIF4E is regulated by the phosphorylation of several serine and threonine residues of 4E-BP. Hypophosphorylated 4E-BP has a strong binding affinity to eIF4E, whereas hyperphosphorylated 4E-BP loses the binding affinity to eIF4E. Indeed, the deletion of 4E-BP significantly increases levels of active eIF4E and enhances the rate of global protein translation, whereas hyposphorylated forms of 4E-BP deplete active eIF4E and thereby inhibit protein translation (Oulhen et al., 2009; Carvalho et al., 2017).

Multiple signaling pathways regulate protein translation by modulating the activity of eIFs (Roux and Topisirovic 2012; Roux and Topisirovic 2018). Especially, mechanistic target of rapamycin (mTOR) is the major signaling pathway involved in regulating the global rate of protein synthesis (Wang and Proud 2006; Ma and Blenis 2009). mTOR is an evolutionarily conserved serine/threonine kinase that stimulates anabolic processes including protein translation, in response to high nutrient/oxygen availability, high energy status, and high levels of insulin/growth factors/growth hormones. mTOR complex 1 (mTORC1), consisting of mTOR, the scaffolding protein raptor, the GTPase β subunit-like protein (GβL), proline-rich AKT substrate of 40 kDa (PRAS40), and Deptor, can directly enhance the activity of translational machinery. For example, the activated mTORC1 hyperphosphorylates and inactivates 4E-BP, which normally attenuates protein translation by preventing eIF4E from interacting with eIF4G to form the eIF4F complex (Wang and Proud 2006; Ma and Blenis 2009).

In addition, mTORC1 can indirectly enhance protein translation by phosphorylating the Thr389 site of the S6 kinase (S6K) and thereby activating S6K (Wang and Proud 2006; Ma and Blenis 2009). S6K has been shown to promote protein translation by phosphorylating multiple components of the translational machinery. For instance, S6K stimulates ribosomal biogenesis and protein translation by phosphorylating ribosomal protein S6 (rpS6) of the 40S ribosomal subunit (Jastrzebski et al., 2007; Chauvin et al., 2014). S6K also disinhibits eEF2 by phosphorylating/inactivating the eEF2 kinase (eEF2K), a well-known negative regulator of eEF2 (Price et al., 1991; Wang et al., 2001). Since eEF2 is a GTPase facilitating the transfer of peptidyl-tRNA from the A site to the P site, S6K activation promotes the ribosomal translocation and ultimately protein translation by stimulating the eEF2 activity. Further, S6K activation upregulates the eIF4A activity by inactivating programmed cell death 4 (PDCD4) and stimulating eIF4B, leading to high protein translation rate (Dennis et al., 2012). PDCD4 is known to repress eIF4A activity by blocking eIF4G-eIF4A interactions (Yang et al., 2003). eIF4B has been shown to bolster the RNA-unwinding activity of eIF4A (Andreou et al., 2017).

Multiple upstream signaling pathways converge on mTOR signaling so that protein translation rate can be adjusted in response to external stimuli and internal cellular conditions (Wang and Proud 2006; Ma and Blenis 2009). Growth hormones and growth factors (e.g. insulin and insulin-like growth factor (IGF)) first activate phosphoinositide 3-kinase (PI3K) *via* receptor tyrosine kinases and associated adaptor proteins such as insulin receptor substrates (IRS). PI3K in turn activates protein kinase B (AKT), which feeds to the mTORC1 signaling *via* tuberous sclerosis complex 2 (TSC2). Growth factors can also stimulate the mTORC1 signaling *via* Ras GTPases and mitogen-activated protein kinase (MAPK) cascades, which also feed to TSC2. Information of the nutrient availability and energy status is conveyed to mTORC1 *via* Rag GTPases, AMPK serine/threonine kinase, and regulated in development and DNA damage responses 1 (REDD1). Via the crosstalk of mTOR signaling with other pathways, cells can timely promote protein translation when environmental conditions are favorable for growth.

Besides mTOR signaling, mRNA processing bodies (known as P-bodies) also play crucial roles in regulating the rate of protein translation in response to environmental stress (Decker and Parker 2012; Luo et al., 2018). P-bodies are cytoplasmic ribonucleoprotein granules known to sequester mRNA and facilitate the mRNA decay *via* deadenylation and decapping, which reduces the mRNA stability. In addition to promoting the mRNA decay, P-bodies can attenuate protein translation rate by trapping translation initiation factors, mainly eIF4E (Brengues and Parker 2007; Rieckher et al., 2018). The P-bodies-dependent halting of global protein translation is particularly beneficial in response to various cellular stresses since this can protect the proteome and divert energy sources into stress responses instead (Yamasaki and Anderson 2008; Rieckher et al., 2018).

## 4 Protein translation declines with age

**FIGURE 3:**
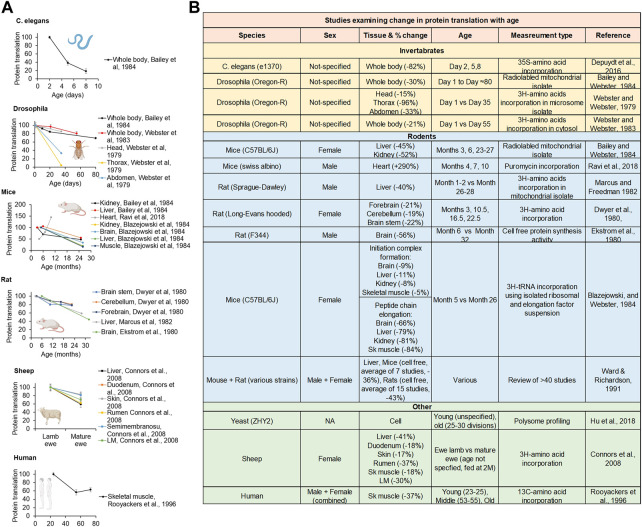
Lifetime protein translation dynamics. **(A)** Replotted protein translation rates across lifespan from studies in *C.* *elegans*, *Drosophila*, mice, rats, and humans. **(B)** Table of studies examining protein translation rates comparing young and old animals. Created with BioRender.com.

Since the 1970s, many investigators have studied how aging impacts the rate of protein translation. In general, the rate of global protein translation is high during early-adulthood but thereafter drops with age (by up to 88%) in yeast, *C. elegans*, *Drosophila*, mice, rats, sheep, and humans (Hrachovec 1969; Young et al., 1975; Webster and Webster 1979; Dwyer et al., 1980; Ekstrom et al., 1980; Webster et al., 1981; Blazejowski and Webster 1983; Webster and Webster 1983; Bailey and Webster 1984; Ward and Richardson 1991; Sonntag et al., 1992; Rooyackers et al., 1996; Connors et al., 2008; Belozerov et al., 2014; Depuydt et al., 2016; Hu et al., 2018; Ravi et al., 2018). This age-related decline in protein translation was observed in a wide variety of cellular fractions (cytosol and mitochondria), tissues, and organs, including brain, lung, heart, thymus, muscle, liver, kidney, intestine, pancreas, etc and has almost universally shown a decline in PT with age this includes a review of >40 studies of protein translation in the liver of mice and rats using multiple measures of protein translation including cell free, liver slices, isolated hepatocytes, and perfused livers (Ward and Richardson 1991) (Figure 3). There is only one study to our knowledge showing PT to increase with age, comparing PT in heart tissue in 4 months–10 month old mice (Ravi et al., 2018). Most of studies examine an older age (22+ Months for the older time point), it is possible that PT rises early life and then subsequently falls. This is consistent with data in livers showing a rise in PT from month three–six then a subsequent decline (Ward and Richardson 1991).

The exact molecular mechanism underlying this age-related decline in protein translation is still unknown. However, several studies suggest that reductions in the activity and levels of eIFs may impair the initiation step and thereby contribute to the age-dependent fall in protein translation. For example, the amount of eIF2 and the activity of eIF2 to promote ternary complex formation decline with age in multiple tissues of rats such as liver, spleen, kidney, lung, and brain (Vargas and Castaneda 1983; Cales et al., 1986; Kimball et al., 1992). Similarly, the amount and activity of eIF2B, which is essential for replenishing the eIF2 activity by facilitating the GDP-GTP exchange, declines with age in brains and livers of rats (Kimball et al., 1992). Interestingly, age-related reductions in levels of active eIF2 were strongly correlated with the decline in protein translation across the lifespan (Kimball et al., 1992). Besides eIF2, the amount of eIF5, which promotes the formation of the 43S pre-initiation complex, also declines with age in several brain areas of rats (Luchessi et al., 2008). Whether the activity and level of other eIFs such as eIF1, eIF3, eIF4A, eIF4E, eIF4G, *etc.* decline with age is still unknown.

The elongation step of protein translation is also compromised with age. For instance, the ribosomal half-transit time (the elongation time required for the synthesis of an average half-length of a nascent peptide) was increased by ∼60% in hepatocytes isolated from old rats (Coniglio et al., 1979). The later *in vivo* studies with yeast, *C. elegans*, mice, rats, and sheep also showed that the rate of peptide elongation significantly slows down with age (Blazejowski and Webster 1984; Merry and Holehan 1991; Connors et al., 2008; Stein et al., 2022). With age, as ribosomal elongation slows down, the frequency of ribosomal pausing increases as well, leading to increased ribosomal collisions and less efficient protein translation (Stein et al., 2022). Based on these observations, subsequent studies have investigated how the amount and function of elongation factors alter with age. The initial study done with *Drosophila* found that the protein level and activity of eEF1A declined with age by ∼60% (Webster and Webster 1983). The decline in eEF1A mRNA level occurred at the same time. Consistent with these data, the catalytic activity of eEF1A to bind aminoacyl tRNA to ribosomes was decreased by 60%–85% in liver, brain, kidney, and skeletal muscle of mice and rats (Moldave et al., 1979; Vargas and Castaneda 1981; Gabius et al., 1983; Webster and Webster 1983; Rattan et al., 1991). Unlike the eEF1 data, there have been some mixed reports on age-related alterations in eEF2. Some studies reported age-related decline in the amount and activity of eEF2 (Takahashi et al., 1985; Munoz et al., 2017), whereas others failed to observe such age-related changes (Moldave et al., 1979; Gabius et al., 1983).

Based on these results, in an attempt to prevent age-related decline in protein translation, Shepherd *et al.* created a *Drosophila* line that overexpresses the eEF1A gene (Shepherd et al., 1989). Interestingly, the extra copy of the eEF1A gene significantly prolonged the median and maximum lifespan of flies (∼41% increase in the median lifespan). However, the later study raised potential problems of the genetic construct and challenged interpretations of the data (Shikama et al., 1994). Shikama *et al.*, just like the original study by Shepherd *et al.*, did observe a robust lifespan extension in the fly line that overexpresses eEF1A. However, Shikama et al. found that the fly line that is supposed to overexpress eEF1A gene did not actually express more eEF1A mRNA or have more eEF1A protein levels. The lifespan extension was proposed to result from not the insertion of the transgene *per se* but the insertion of *P*-element reverting a life-shortening effect of the *rosy* gene mutation background. Since then, no studies have investigated how age-related alterations in eEF1A impact protein translation and lifespan.

The ribosomal loading to mRNA is also impaired with age. For example, the proportion of ribosomes in polyribosome (polysome) fractions significantly decreases with age in multiple animal species (Wallach and Gershon 1974; Kurtz 1978; Webster et al., 1981; Pluskal et al., 1984; Lee et al., 1993; Hu et al., 2018). Conversely, there is an age-dependent increase in monomeric ribosomes (monosomes), implying that less ribosomes are actively associated with mRNA with increasing age. As a result, the ability of ribosomes to incorporate labeled amino acids into a polypeptide chain is severely compromised with age (Britton and Sherman 1975; Kurtz 1978; Mori et al., 1979; Ekstrom et al., 1980; Linnemann et al., 1993). The age-related reductions in the amount and functionality of various eIFs (discussed above) may contribute to the impairment in ribosomal attachment to the mRNA. Another contributor to the compromised ribosomal loading would be age-related decline in translational output of ribosomal protein mRNA and transcripts related to ribosomal biogenesis, potentially due to their increased methylation (D'Aquila et al., 2017; Hu et al., 2018; Anisimova et al., 2020). In fact, ribosome occupancy of translation-related genes drops with age by twofold or even more (Anisimova et al., 2020). At the same time, translational output of genes that downregulate protein translation is increased: e.g. *ssd1*, which sequesters mRNAs and translational machineries into P-bodies and *Gcn2*, which inactivates the eIF2-dependent ternary complex formation (Hu et al., 2018).

Recent studies suggest that not only reductions in ribosomes or translational machineries *per se* but also the disruption of the stoichiometry of translational machinery components may contribute to decline in protein translation with age. For example, the parallel analysis of Ribo-seq (ribosome profiling) data and RNA-seq data in young and old yeast cells showed that the abundance of transcripts increased with age whereas the translation of each transcript decreased with age (Hu et al., 2018; Tye et al., 2019). These data suggest that the imbalance between transcripts and translational machineries becomes exacerbated with age, which may cause inefficient loading of translational apparatus to mRNA. Similar divergent changes in transcripts and proteins with age were observed in mammals as well. For example, in African killifish, levels of transcripts encoding ribosomal proteins continuously increased with age whereas the abundance of ribosomal proteins decreased with age (Kelmer Sacramento et al., 2020). This age-related loss of stoichiometry of ribosomal proteins may impair the ribosomal assembly, leading to decreased efficiency of protein translation. The subsequently increased pool of orphan ribosomal proteins may increase the risk of protein misfolding and aggregation (Tye et al., 2019), which may further attenuate protein translation by activating the PERK-UPR axis (Walter and Ron 2011). Consistent with these results, the translational output of ribosomal proteins increased whereas that of other translational factors decreased in rat brains across ages (Ori et al., 2015). This proteomic imbalance between ribosomal proteins and translational factors may impair efficient assembly of pre-initiation complex and compromise the ribosomal loading to mRNA.

## 5 Life-long reduction in protein translation improves lifespan and healthspan

**FIGURE 4:**
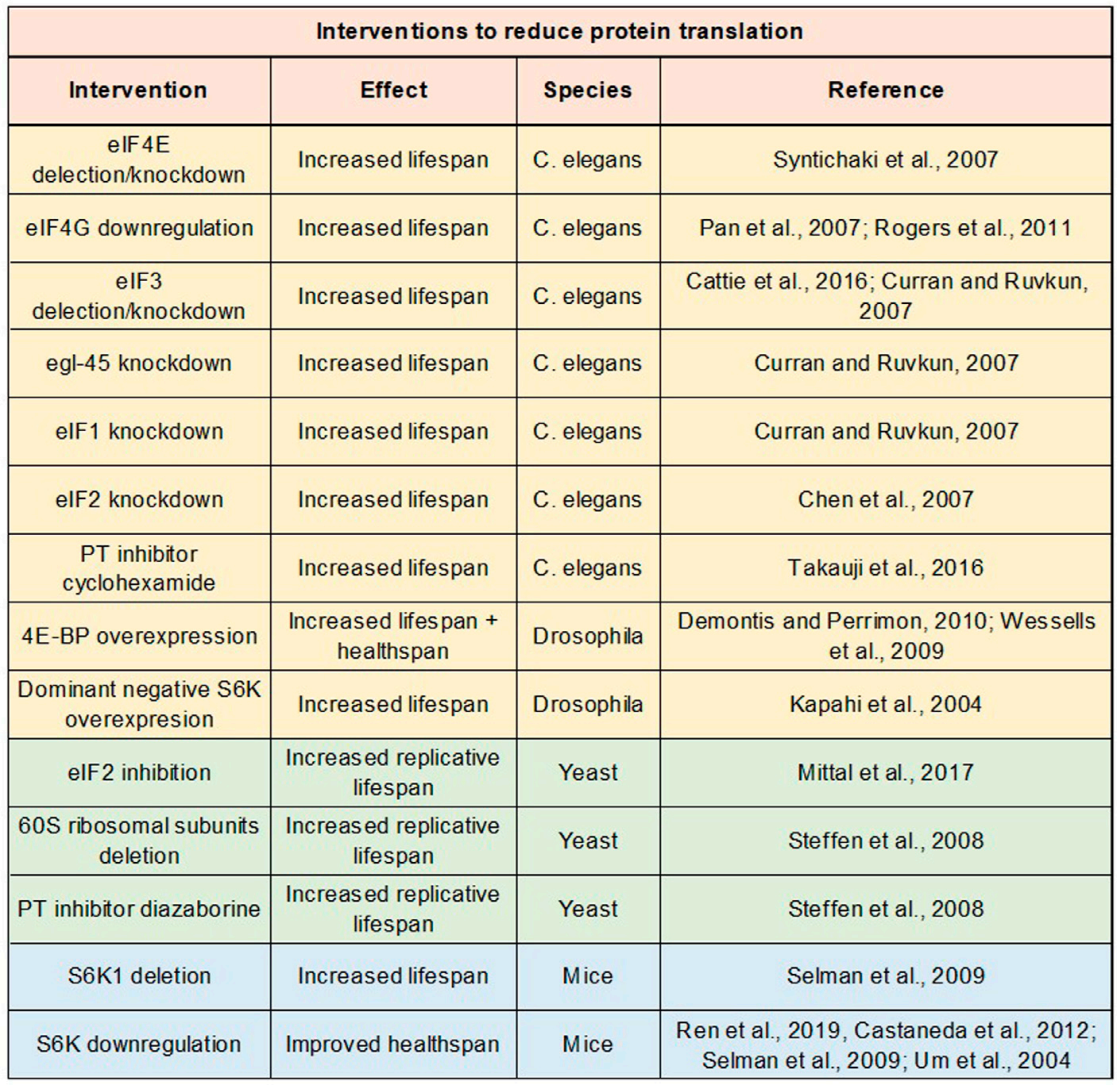
Impact of protein translation repression on lifespan and healthspan. Table of studies examining impact on lifespan or healthspan of genetic and drug interventions to repress protein translation.

Protein translation is an essential cellular process, playing crucial roles in growth, development, and reproduction (Pan et al., 2007). As previously discussed, protein translation precipitously declines with age, staying at low basal levels throughout middle-old ages in multiple animal species, including humans. One would expect that lowering protein translation would be detrimental to health. However, a life-long reduction in protein translation slows down aging, prolongs lifespan, and ameliorates cellular senescence and several age age-related diseases (Scheuner et al., 2005; Pan et al., 2007; Syntichaki et al., 2007; Steffen et al., 2008; Selman et al., 2009; Tain et al., 2009; Blagden and Willis 2011; Rogers et al., 2011; Martin et al., 2014; Takauji et al., 2016; Ren et al., 2019) (Figure 4).

From 1970s to 1990s, most of aging studies had focused on the effects of aging on protein synthesis. Since the early 2000s, investigators began to examine the reverse relationship: the effects of protein synthesis on the aging process. Syntichaki *et al.* were one of the first groups to study how perturbations in protein translation impact the animal lifespan (Syntichaki et al., 2007). Syntichaki *et al.* attenuated protein translation in *C. elegans* by deleting or knocking down the translation initiation factor eIF4E isoforms in somatic tissues. This manipulation substantially prolonged the nematode lifespan (∼55% extension in the median lifespan). Subsequent studies inhibited mRNA translation by mutating or knocking down other translation initiation factors and observed robust lifespan extension effects as well. For example, downregulation of eIF4G, which interacts with eIF4E to facilitate formation of the eIF4F complex, improved the nematode lifespan by more than 30% (Pan et al., 2007; Rogers et al., 2011). Similarly, deletion or RNAi of eIF3, which plays a critical role in formation of the 43S pre-initiation complex, led to ∼40% extension of the *C. elegans* lifespan (Curran and Ruvkun 2007; Cattie et al., 2016). Knocking down other translation initiation factors such as eIF1, eIF2, eIF2B, eIF4A, eIF5A, *etc.* also significantly improved the lifespan (Hamilton et al., 2005; Chen et al., 2007; Curran and Ruvkun 2007; Tohyama et al., 2008).

Later studies have targeted different translational machineries to inhibit protein synthesis in animal models other than *C. elegans*. Similar lifespan extension effects were observed. For example, overexpression of 4E-BP, which sequesters eIF4E and thereby attenuates protein translation, ameliorated age-related protein aggregation, improved functions of heart and skeletal muscle at old ages, and extended the *Drosophila* lifespan (Wessells et al., 2009; Demontis and Perrimon 2010). Moreover, reducing protein translation by inhibiting the eIF2 activity *via* Gcn4 extended the replicative lifespan of *S. cerevisiae* (Mittal et al., 2017). Further, deletions of or mutations in several ribosomal proteins significantly increased the lifespan of the budding yeast and filamentous fungi (Belcour et al., 1991; Steffen et al., 2008). Likewise, reducing the levels and activity of S6K, which promotes ribosomal biogenesis, robustly improved the lifespan of *C. elegans*, *Drosophila*, and rodents (Kapahi et al., 2004; Pan et al., 2007; Selman et al., 2009). For example, in fruit flies, dominant negative forms of S6K significantly increased the lifespan, whereas constitutively active forms of S6K caused accelerated aging and shortened the lifespan (Kapahi et al., 2004). Consistent with these results, inhibiting mTOR signaling, which enhances protein translation *via* S6K activation, promotes longevity in multiple animal models (Johnson et al., 2013).

Pharmacologically inhibiting protein translation has also been shown to slow down aging as well. For example, cyclohexamide, a well-known protein synthesis inhibitor targeting the elongation phase of translation, abolished senescent features in human cells and robustly improved the *C. elegans* lifespan (Takauji et al., 2016). In addition, diazaborine, which reduces levels of 60S ribosomal proteins and inhibits ribosomal biogenesis and maturation, significantly increased the replicative lifespan of the budding yeast (Steffen et al., 2008). Further, attenuating protein translation by minocycline enhanced proteostasis and promoted the longevity in *C. elegans* (Solis et al., 2018). Minocycline also had neuroprotective and anti-inflammatory effects and ameliorated AD-related cognitive deficits in rodents (Choi et al., 2005; Festoff et al., 2006; Seabrook et al., 2006; Choi et al., 2007; Noble et al., 2009). However, unlike cyclohexamide and diazaborine, minocycline is an antibiotic that does not specifically target protein translation and has the potential to impact other pathways.

Manipulations that lower protein translation improve not only lifespan but also healthspan and ameliorate several age-related diseases, including frailty, osteoporosis, metabolic disorders, cardiovascular disease, cancer, and neurodegenerative disorders. For example, diminishing protein translation rate by inhibiting ribosomal biogenesis alleviated physiological aging of human mesenchymal stem cells and counteracted the development of frailty and osteoarthritis in mice (Ren et al., 2019). Moreover, downregulating S6K signaling to attenuate protein translation in mice improved the locomotor activity, increased the population of naïve T cells, enhanced the bone volume and density, and protected against age- and diet-induced obesity/diabetes by improving insulin sensitivity and reducing adiposity (Um et al., 2004; Selman et al., 2009; Castaneda et al., 2012). Consistent with these studies, in rodent models of type II diabetes, S6K signaling was overactive and protein translation rates were elevated in islet β-cells (Hatanaka et al., 2014; Talaei et al., 2014). In addition, enhancing protein translation *via* overactivation of eIF2 impaired glucose tolerance and caused diabetic phenotypes in mice (Harding et al., 2001; Zhang et al., 2002b). Conversely, attenuating protein translation by lowering the eIF2 activity ameliorated the apoptosis of β cells and improved the glucose tolerance (Scheuner et al., 2005; Back et al., 2009).

Further, reduction in protein translation by depleting eIF4E with 4E-BP slowed down cardiac aging and decreased the frequency of cardiac failure at old ages (Wessells et al., 2009). Knocking down eIF4E ameliorated tumorigenesis as well, and consequently, drugs targeting eIF4E to inhibit mRNA translation have been heavily tested in clinical trials for hematologic malignancies (Rinker-Schaeffer et al., 1993; Blagden and Willis 2011). Overexpression of 4E-BP also prevented dopaminergic neuronal loss and ameliorated locomotor deficits in fly models of Parkinson’s disease, induced by *parkin* and *pink1* mutations (Tain et al., 2009; Liu and Lu 2010). Likewise, in another fly model of Parkinson’s disease caused by the mutation in leucine-rich repeat kinase 2 (LRRK2), the most common genetic cause of both familial and sporadic Parkinson’s disease, global protein translation rate was elevated due to phosphorylation of ribosomal protein s15 (Martin et al., 2014). Introducing phosphodeficient s15 to attenuate protein translation ameliorated dopaminergic neuronal degeneration and age-related locomotor deficits in LRRK2 mutant flies (Martin et al., 2014).

A few studies suggest that overactive protein translation may contribute to the AD pathogenesis as well. For example, the pathogenic Aβ_1-42_ increased protein translation by activating the eIF4E activity (Ghosh et al., 2020). In addition, cytoplasmic FMR1-interacting protein (CYFIP), which represses protein translation by blocking eIF4E-eIF4G interactions, was downregulated in post-mortem brains of AD patients, and CYFIP reduction caused AD pathologies in mice (Tiwari et al., 2016; Ghosh et al., 2020). Further, tau K174 acetylation, which occurs in brains of AD patients/mice at early stages of the disease, enhanced protein translation by causing nucleolar expansion (Min et al., 2015; Portillo et al., 2021). Reduction in protein translation by downregulating S6K signaling improved spatial memory and synaptic plasticity in an AD mouse model (Caccamo et al., 2015).

Consistent with the data discussed above, long-lived strains of animals show low rates of protein translation. For example, long-lived *C. elegans* strains with reduced insulin/IGF signaling, reduced mTOR signaling, reduced germline signaling, or impaired mitochondrial electron transport chain functions, *etc.* showed smaller sized nucleoli, decreased ribosomal biogenesis, and significantly lower protein translation rates (Depuydt et al., 2013; Tiku et al., 2017). Likewise, long-lived growth hormone-deficient Snell dwarf mice exhibited decreased rates of protein translation in liver, heart, and skeletal muscle (Bates and Holder 1988; Thompson et al., 2016). Conversely, fibroblasts from patients with premature aging disorders showed nucleolar expansion, increased ribosomal biogenesis, and elevated protein translation rates (Buchwalter and Hetzer 2017). These data indirectly suggest that long-lived animals may achieve the longevity by sustaining low rates of protein translation.

## 6 Discussion and conclusion: Protein translation paradox

Extensive studies from the 1970s to 1990s have shown protein translation rates to decline over the course of age. These studies encompass a menagerie of organisms including yeast, *C. elegans*, *Drosophila*, mice, rats, sheep, and humans in a plethora of tissues including brain, lung, heart, thymus, muscle, liver, kidney, intestine, pancreas, *etc.* Thus, it has been postulated that reduction in protein translation would accelerate the aging process. However, research since the 2000s has shown that life-long reduction in protein translation actually robustly improves the lifespan and healthspan. These conflicting findings raise the so-called *protein synthesis paradox* (Blagosklonny et al., 2007). The paradox has not been resolved, which imposes a substantial barrier to fully understand how protein translation regulates aging.

The paradox may have mainly arisen from the assumption that age-related decline in protein translation is a *passive* byproduct of aging. Proteasomal and autophagial degradations decline with age, so in order to match with the reduced capacity of degradation machineries and thus maintain proteostasis, protein translation may have been repressed with age as an *adaptive* response. In this way, organisms can minimize proteostatic burden, reduce levels of damaged and aggregated proteins, and thus optimize the lifespan. This new theoretical framework can resolve inconsistently-seemed findings with regard to translational regulation of aging. To validate this idea, further studies should be done to investigate how the rise and fall in protein translation across lifespan regulate aging and proteostasis. Till now, all the aging studies have manipulated protein translation throughout the whole life rather than modifying it in a specific time window. With understanding of how lifetime protein translation dynamics regulate the onset of aging, we will be able to fully appreciate how protein translation modulates the aging process.
